# Configuring the coupling and deparadoxization of law and politics: a framework for a systems-theoretical analysis of new Latin American constitutionalism

**DOI:** 10.3389/fsoc.2026.1886728

**Published:** 2026-08-06

**Authors:** Franz Xavier Barrios-Suvelza, André Brodocz, Hannah Vermaßen

**Affiliations:** Faculty of Economics, Law, and Social Sciences, University of Erfurt, Erfurt, Germany

**Keywords:** constitution, deparadoxization, functional differentiation, new Latin American constitutionalism, Niklas Luhmann, structural coupling

## Abstract

This article advances a systems-theoretical framework and methodology for analyzing the contingent configurations of law, politics, and the constitution in modern society. Departing from Niklas Luhmann’s model of functionally differentiated, operationally closed systems, it addresses the problem of how structural coupling and deparadoxization between law and politics vary under different conditions of functional differentiation. The study adopts a theoretically oriented, reconstructive approach that integrates and critically compares existing systems-theoretical accounts, particularly those of Marcelo Neves and Aldo Mascareño. As key theoretical contribution, the article suggests six potential configurations of law, politics, and the constitution that go beyond Luhmann’s well-known account of the symmetric structural coupling of law and politics through the constitution in Western liberal democracies: symmetric, asymmetric, pure, alternative, and volatile coupling, as well as dedifferentiation. The contingency from which the differences between the six distinct configurations of law, politics, and constitution can be derived is systematically situated within Luhmann’s systems theory at the various levels of systemic operations, systemic self-descriptions, and system differentiation. As a key methodological contribution, the article argues that any empirical operationalization derived from this must therefore be based on research questions specific to each of the three levels and identifies different empirical data and methods required at each of these levels. These requirements are specified in the empirical context of the new Latin American constitutionalism in Bolivia and Ecuador, where the caesuras of plurinationality, the elevation of the people and the legal subjectivation of nature indicate fundamental shifts in the differentiation and coupling of law and politics.

## 1 Introduction

According to Luhmann (1997, 707ff.), the development of modern society follows the principle of the functional differentiation of operationally closed systems. Like religion, science, and the economy, politics and law have thereby differentiated into autonomous function systems, each with its own binary code. All function systems have become structurally coupled with one another through various institutions. In Luhmann’s account, the structural coupling of the legal system and the political system through the constitution has a distinctive status. Unlike structural couplings between other function systems, the constitution serves law and politics not only by keeping them mutually irritable; both systems also deparadoxize themselves reciprocally through the constitution (Luhmann, 2000, 69ff., 388ff.).

This specific configuration of law, politics, and the constitution, which primarily follows the development of the Western liberal-democratic constitutional state (Greven, 2001, 210), is not, however, compelled by systems theory. Whether this configuration takes the form of a structural coupling and reciprocal deparadoxization of law and politics depends instead on the “history of differentiation” and is thus historically variable (Luhmann, 2000, 369; see also Luhmann, 1988, 154).^1^ Luhmann (1990, 210) even regards this specific configuration of law, politics, and the constitution as an “evolutionary achievement,” because both systems use the constitution for structural coupling and, at the same time, for deparadoxization.

As Giancarlo Corsi (2016b, 261) points out “[i]t is no coincidence […] that Luhmann places the constitution within an evolutionary process” because this “enables him to disregard its inventors’ intention: in other words, the constitution is the result of a process that has manifested its potential in a way that goes well beyond those intentions,” on the one hand. On the other hand, the fact that evolution can take other directions has also been emphasized repeatedly in the theoretical debate following Luhmann (see Febbrajo and Corsi, 2016). Mathias Albert, for example, observes an increasing differentiation of the political system and the legal system in world society. This is driven by the “declining efficacy of a territorial-segmentary internal differentiation” (Albert, 2002, 71) of both systems. On the side of the political system, this differentiation leads, particularly through transnational migration, to a weakening of territorial boundaries for the construction of collective identities (Albert, 2002, 202; see also Nassehi, 2002). The legal system differentiates internally into various sectors, among which state legal orders constitute only one sector among others, especially transnational and international legal orders (Albert, 2002, 232 f.). Albert (2016, 6 f., 81ff.) likewise argues that world politics has become internally differentiated. He distinguishes between a system of states and a system of world politics. This differentiation is accompanied by the growing importance of semantic references to the “global,” the “world,” and the “international” (Albert, 2016, 126ff.).

The fact that “the system boundaries of the legal system no longer coincide with those of the political system” (Albert, 2002, 211; similarly, Willke, 2019, 19ff.) opens up novel perspectives for deparadoxization for both systems, which have so far been studied above all for transnational and international legal orders. Gunther Teubner and Andreas Fischer-Lescano have made especially important contributions here. Teubner (2015, 79ff.) has shown how the legal system is also used by nonpolitical function systems, such as the economy or science, to externalize their paradoxes in the form of “civil constitutions.” However, this only succeeds with “varying intensity”, as a “full symmetry of reciprocal paradox externalization” has only developed between the political and legal systems so far (Teubner, 2015, 84). That the legal system nevertheless decouples from the political system at least in certain sectors is also shown by justice semantics that transcend the legal system itself (Teubner, 2013, 345ff.). In the legal system of world society, this leads to a fragmentation of legal orders whose own rationalities collide with increasing frequency (Fischer-Lescano and Teubner, 2006). This contributes to the fact that the system of world politics, as a subsystem of the political system in world society alongside nation-state subsystems, begins to couple structurally with the world legal system, as a subsystem of the legal system in world society alongside other state-legal as well as sectoral legal orders. This is achieved by means of a constitution of its own beyond state constitutions, the “global constitution” that is emerging from various legal sources, customs, and practices, and whose validity has found its essential symbol in human rights (in detail Fischer-Lescano, 2005).

Yet systems theory has so far scarcely investigated empirically how law, politics, and the constitution may also configure themselves differently specifically in the context of the territorial-segmentary internal differentiation of the legal system and the political system—in short, at the regional and nation-state level. The pioneering approaches of Marcelo Neves and Aldo Mascareño are an exception. They have so far produced the most important systems-theoretical studies on Latin American states (see Cortés Morales, 2014; Calise, 2024) and are therefore presented in detail in the next section.

The variance of potential configurations, however, has still not been sufficiently captured. We argue that systems theory allows for at least six possible configurations of law, politics, and the constitution. Each differs in how law and politics are coupled and how they deparadoxize themselves through the constitution: First, law and politics may be constitutionally coupled and deparadoxize themselves symmetrically through the constitution (“symmetric coupling”); second, only one of the two systems may use the constitution for deparadoxization (“asymmetric coupling”); third, law and politics may be constitutionally coupled without using the constitution for deparadoxization (“pure coupling”); fourth, the two systems may be coupled through social instances other than the constitution (“alternative coupling”); fifth, different forms of coupling may emerge only temporarily and remain unstable (“volatile coupling”); finally, law and politics may fail to differentiate into two operationally closed function systems (“dedifferentiation”).

The aim of this article is not to provide confirmation of these hypotheses to be examined in the course of our investigation, but rather to outline the main features of the research design to be used in future studies to answer these questions. In what follows, we will therefore first set out how law, politics, and the constitution are configured according to the approaches of Neves and Mascareño (2). We then explain why at least six different variants of the configuration can be distinguished in systems-theoretical terms and with which methods and materials they can be empirically reconstructed (3), before finally discussing how the approaches of Neves and Mascareño can be systematically located within this variance (4).

## 2 Systems-theoretical perspectives on law, politics, and constitution in Latin America

### 2.1 Marcelo Neves and the critique of the primacy of functional differentiation in Latin America

For Marcelo Neves, the primacy of functional differentiation in modernity assumed by Luhmann has prevailed only in a few states of the Global North. Neves (2012, 19) therefore speaks of a “bifurcation” into central and peripheral paths in the “development of modern (world) society,” and this also applies to Latin America in comparison with what O’Donnell (2001, 7) calls the “northwestern quadrant of the world.” Neves retains the feature of modernity for Latin America but relativizes it by referring to it as “peripheral” modernity. For Neves, the branch of this bifurcation that does not yet exhibit fully developed functional differentiation, and in which Latin America is to be located analytically, remains part of the development of modern world society, albeit in a strongly modified form.

For Neves, Latin America is characterized by the coexistence of different forms of social differentiation. In peripheral modernity, this constellation assumes heterarchical traits, since functional differentiation does not possess primacy there. Neves (2012, 24) therefore speaks of a “mixed form” of social differentiation. This does not rule out the possibility that, despite the absence of the primacy of functional differentiation in Latin America, a variant of expectation stabilization can emerge at least at the interactional and organizational level. While Neves’s concepts of bifurcation and mixed forms operate at the level of system differentiation, he also examines the configurations that emerge from specific forms of linkage between different functional systems, even under conditions in which functional differentiation does not hold primacy.

Neves’s thesis is that no functional system in Latin America has undergone autonomous development. He attributes this blocked development of the function systems to the absence of the primacy of functional differentiation there. Instead, he diagnoses an economically conditioned bifurcation into a central and a peripheral modernity (Neves, 2006, 248–249), which “affects the reproduction of all social systems” and applies “above all to law and politics as state-organized systems.” This means that the legal system operates not autopoietically but mostly “allopoietically”; that is, it does not reproduce itself but relies on the “criteria, programs, and codes of its environment” (Neves, 2003, 258). In this context, Neves (2004, 177) notes that, although no autonomous legal sphere has emerged in Latin America, an identity of law can nevertheless be seen, at least “at the level of normative textual structures”; but this identity is immediately “gradually destroyed or distorted in the process of legal concretization” (Neves, 2004, 177). According to Neves (2004, 170), the differentiation of an autonomous legal system is blocked above all by the political system and the economic system. For Neves (2012, 21; 1998, 122ff), this produces a “confusion” of the codes proper to each function system. According to Neves (2004, 170), Latin America, as a peripheral region, faces a veritable tangle of codes even within the legal system. He then speaks of “mutually destructive relations” and of the operative “lack of distinction among the different legal domains” (Neves, 2004, 170).

Neves (2006, 260) does not limit his diagnosis of incomplete functional differentiation to law but extends it to politics as well. For “the subordination of law to politics implies neither autonomy nor a strong identity of the political system” (Neves, 2006, 261). Elsewhere, he ultimately denies functional differentiation as an operationally closed system not only to law, but also to politics in the case of peripheral modernity; electoral fraud, for example, is evidence of the “breach of the operational closure of the political system” (Neves, 2003, 264).

### 2.2 Aldo Mascareño and the concentric structure of functional differentiation in Latin America

In contrast to Neves, Mascareño (2012, 78) adheres to the idea of the primacy of functional differentiation in Latin America, as an expression of this primacy in world society. Mascareño’s (2012, 45) thesis is that functional differentiation exists “in all regions of the world”; it is merely implemented differently depending on regionally specific conditions. He argues that the primacy of a form of system differentiation in Latin America is by no means new. In the colonial period, for example, the variant of stratificatory differentiation had priority. It prevailed over segmentary and center/periphery variants. Mascareño emphasizes that his thesis does not imply the homogenization of world society (Mascareño and Chernillo, 2009, 90). This is because both functional differentiation and cosmopolitan principles constantly encounter semantic particularisms and local structures in the different regions of the world. Even if this does not call modernity in Latin America into question, it does point to different paths through modernity (Mascareño and Chernillo, 2009, 91).

Although Mascareño (2000, 35) acknowledges the existence of functional differentiation in Latin America, he does not recognize the same polycentric configuration that occurred in Europe. Mascareño (2000, 27) bases his thesis on a historical overview of the course of functional differentiation in Latin America, by means of which he traces this non-polycentric development. The non-polycentric dynamic had already emerged in the nineteenth century in the form of a centralized state. This primacy of the state is still evident in state interventions into the economic system, as they were expressed most clearly at the beginning of the twentieth century in the struggle against urban poverty (Mascareño, 2000, 28). According to Mascareño, this gives rise to a “concentric” structure characterized by the primacy of one or more function systems within functional differentiation. In contrast to the developed West, functional differentiation in Latin America therefore took place “concentrically” (Mascareño, 2000, 19).

Mascareño’s thesis of a “concentric” form of functional differentiation established in Latin America points to a hierarchical situation in which one function system – here, the political system – assumes a superior role, particularly in relation to law. Mascareño, however, understands this concentricity as a historically contingent result shaped by the populist semantics of *pueblo* (Mascareño, 2018, 48) but whose development is not yet complete (Mascareño, 2000, 29). To be sure, in Latin America, the function systems of education and art, but above all the economic system, have meanwhile gained autonomy and, in this form, are already opposing the otherwise dominant political sphere.

Because Mascareño (2012, 20) assumes the primacy of functional differentiation in Latin America, albeit in a different “structure” than in Western Europe, he does not, like Neves, speak of a “mixed form” at the level of system differentiation. Mascareño’s approach thus occupies an intermediate position between Luhmann and Neves. He shares Luhmann’s view that functional differentiation is the defining feature of modernity, including in Latin America. However, with his thesis of concentrically structured functional differentiation in Latin America, he relativizes the necessity of the evolution of the developed West without rejecting functional differentiation entirely, as Neves does.

Since Mascareño (2000, 30) assumes that the function systems in Latin America are increasingly able to close themselves operationally and differentiate stably as autonomous function systems (as the development of the economy and art shows), a future assertion of polycentric functional differentiation in Latin America does not appear entirely implausible to him. Mascareño (2000, 30, 36; 2003, 9), however, doubts that the path toward this functional differentiation in Latin America is predetermined. The concentric variant should therefore not be considered as an “abnormality” (Mascareño, 2003, 15). Moreover, he considers that social processes today may destabilize the concentric form and, given the higher complexity of our time, could lead to polycentric functional differentiation (Mascareño, 2003, 20). More likely, according to Mascareño (2003, 20), is the emergence of a “post-concentric” society that will likewise differ from polycentric development according to the European model.

It is not clearly discernible in Mascareño whether this predominant role of politics, which according to his thesis underlies the concentric form of functional differentiation (Mascareño and Chernillo, 2009, 87; Mascareño, 2012, 20), presupposes an autonomous development of this function system. At one point Mascareño (2012, 24) suggests that the legal system in Latin America was attacked immediately after independence by “autonomous operations of the political system.” However, elsewhere, Mascareño (2000, 27) expresses regret that all functional systems in Latin America are prevented from developing autonomously, which he attributes to the concentration of social communication within certain systems at the expense of others.

In fact, Mascareño’s (2003, 4) approach proceeds from autonomous, semi-autonomous, and non-autonomous function systems. Even a system into which another system intervenes can therefore remain “partly autonomous” (Mascareño, 2012, 25). Mascareño (2000, 26) thus points to an asynchronous development of the autonomy of the function systems and in this way seeks to relativize Luhmann’s assumption of a “spontaneous” autopoietic dynamic. Accordingly, unlike Luhmann, Mascareño (2000, 29) prefers to speak of lower “degrees of autonomy” rather than of “no autonomy.” Elsewhere, however, Mascareño (2003, 2) is more skeptical about the autopoietic constitution of autonomous systems in the case of Latin America. According to him, there is no system reproduction in Latin America that proceeds exclusively autopoietically (Mascareño, 2003, 15).

Mascareño therefore adopts a dynamic view of system differentiation. What appear to be cases of dedifferentiation are, in his view, only temporary episodes. These interventions may restrict systemic autonomy for a limited time, but they do not suspend the primacy of functional differentiation (Mascareño and Chernillo, 2009, 88). These episodes “can be described as systemic interventions which, although they exclude the strictly autonomous functioning of a system, are not capable of completely grasping the other systems spatially and temporally” (Mascareño and Chernillo, 2009, 88). From this perspective, Neves’s very skeptical view of the assertion of functional differentiation appears misguided. First, because what Neves regards as the rule (no autonomous system) occurs only sporadically and, second, because when it does occur, it never does so in an absolute manner. According to Mascareño (2012, 29), an important consequence of this dynamic is the disappointment of expectations and the uncertainty of communicative connections: “One does not know whether one will obtain justice, even when the legal decision is already in place.” The instability of expectations thus becomes stable in its own right when informal expectation structures emerge and collide with “the expectation structures of a functionally differentiated society” (Mascareño, 2012, 33). Finally, it should be noted that Mascareño does not causally attribute this development of functional differentiation in Latin America to politics alone. He also mentions the economy as a strengthened system, although in his view the economy itself is overrun by politics.

### 2.3 Comparing the approaches of Neves and Mascareño

The two approaches of Neves and Mascareño already show how differently law, politics, and constitution can be configured from a systems-theoretical perspective when the empirical and historical reference shifts from Western Europe to Latin America. Both make clear that Luhmann’s configuration of law, politics, and the constitution in the form of the Western liberal constitutional state is only one variant. Despite a similar reference, the two approaches also arrive at different variants because they provide fundamentally different answers to two questions: the question of the differentiation of law and politics as operationally closed and autonomous function systems, and the question of the role of the constitution in this configuration.

Mascareño (2000, 27) sees “systems with specific functions” which can obviously be reconstructed in Latin America independently of whether and when functional differentiation historically established itself. According to Mascareño (2003, 17), one can therefore assume that there is at least a “minimal stability” of the systems until the corresponding organizations are formed, even if functional differentiation has otherwise not really developed. Mascareño speaks in this context of the formation of “systemic infrastructures.” In short, “politically based expectation structures and an organizational, political infrastructure” emerge (Mascareño, 2003, 91). One can furthermore speak of at least existing “soft operations” in the system (Mascareño, 2012, 39). Even more, Mascareño holds that self-referentiality is already effective without the stage of autopoiesis having been reached (Mascareño, 2012, 40).

Although Neves, like Mascareño, does not exclude functional specialization, he sees system autonomy of law and politics in Latin America as scarcely developed. According to Neves, however, the incomplete functional differentiation of law is nonetheless implicated in the functional differentiation of politics. In his view, both systems appear to function in an asymmetric dynamic in which one system (politics) needs the other (law) in order to differentiate itself. Neves (2006, 252) emphasizes that the autonomization of the political system can only occur if law becomes relevant as the bearer of a second coding: without the incorporation of the legal code into the political system, the latter would not be able to defend itself against “particularistic pressures and immediate factors of its environment.” Through the constitution, politics as a system thus obtains a “legal immunization incorporated into the construction of its own autonomy” (Neves, 2006, 254).

For Mascareño, Neves’s rejection of functional differentiation goes too far. In Latin America, rather, only recurrent episodes of dedifferentiation occur (Mascareño, 2000, 35). These episodes are indeed an obstacle to the autonomy of the function systems, but never so extensive as to allow the primacy of functional differentiation to be disputed (Mascareño and Chernillo, 2009, 87). For if the differentiation of the function systems itself were only episodic, Mascareño could not consistently assert any form of functional differentiation at all. He explains this development in Latin America by means of his thesis of a particular concentric structure of functional differentiation established there. For Mascareño (2003, 2), the empirical predominance of politics in Latin America appears in its relation to law in the frequency of states of exception and in its relation to the economy in the pursuit of a strategy of import substitution. According to Mascareño (2012, 98), the economy appears as an autonomous function system in Latin America only once it has achieved operational closure, as the liberalization of economic processes began and it resisted state interventionism. Measures aimed at a systemic differentiation of the economy are, as a rule, associated with large-scale privatization programs (Mascareño, 2012, 99). Mascareño describes the increasing operational closure of systems generally as proceeding along four cycles, which he takes over from Teubner: from self-observation/self-description (Z1), through self-organization/self-regulation (Z2) and reproduction (Z3), to autopoiesis itself (Z4). According to Mascareño, in twentieth-century Latin America both politics (Mascareño, 2012, 93) and the economy (Mascareño, 2012, 99) reach the “fourth cycle,” which includes autopoiesis. Only law, according to Mascareño, still remains behind this point today.

A second reason for the different variants advanced by Neves and Mascareño has to do with the question of the role of the constitution. Neves (2003, 264) takes the view that the allopoiesis of law in peripheral modernity is the result of a failure of the constitution. According to Neves (2004, 183), the constitution in Latin America was “deformed” from the beginning. This condition resulted from the fact that the constitution was distorted “during the process of concretization by virtue of being overlaid by particularistic political pressures and specific economic interests” (Neves, 2004, 183). One might object, by contrast, that the weakness of law in Latin America was, inversely, a precursor of the insufficiency of the constitution.

For Neves (2003, 265), the weakness of the constitution in Latin America is shown by the absence of a “constitutional normativity attributable to the constitutional text.” Nevertheless, Neves does not consider the constitution meaningless for that reason. In any case, it has a “political-discursive function” (Neves, 2004, 184). Correspondingly, Neves (2006, 265; 1998) also speaks of the “hypertrophied political-symbolic role of the constitution” in Latin America. A constitution of this quality, however, makes structural coupling impossible according to Neves (2006, 254). By contrast, Mascareño and Chernillo (2009, 92) speak of the “emergence of structural couplings” in Latin America despite the particular form of modernity in this region. At another point, however, Mascareño (2012, 27), with respect to Latin America, declares the state, and not explicitly the constitution, to be the “formal connection between political and legal procedures” during the nineteenth and twentieth centuries.

In this respect, Neves’s argument corresponds to Luhmann’s few observations on the configuration of law, politics, and the constitution beyond Europe. At one point, Luhmann states that in “many developing countries” constitutions “serve almost only as instruments of symbolic politics, because it has not yet been possible to close the legal system operationally” (Luhmann, 1993a, 478). Nevertheless, he emphasizes that “even then […] the modern pattern of structural coupling is still recognizable, even if only as true (that is, functioning) semblance.” At first sight, this looks as though Luhmann recognizes structural coupling even when the legal system is not operationally closed and the constitution operates more symbolically, that is, as “semblance.” But Luhmann (1993a, 478f) immediately clarifies: “In the full sense, however, the evolutionary achievement ‘constitution’ fulfills its function only under the condition of functional differentiation and operational closure of the political system and the legal system.”

## 3 Theoretical framework for the analysis of law, politics, and constitution

### 3.1 Hypotheses: six different configurations

The configuration of the legal system and the political system in the functionally differentiated society is determined by how the two systems structurally couple and deparadoxize themselves. Structural couplings become necessary as soon as both systems differentiate as function systems through operational closure (Luhmann, 1993a, 440ff.). They designate the relations that a system, under these conditions, enters into with other systems in its environment when it “permanently presupposes certain peculiarities of its environment and relies structurally on them” (Luhmann, 1993a, 441). For the legal system and the political system, as already explained in the introduction, the constitution serves according to Luhmann as structural coupling. In the legal system, the constitution structures the legal form in which political decisions are communicated in the legal system, while in the political system the constitution structures the legal possibilities for enforcing collectively binding decisions by means of laws (Luhmann, 1993a, 470ff.).

In addition, both systems deparadoxize themselves reciprocally through the constitution. According to Luhmann, the problem of deparadoxization arises for function systems when the binary code constitutive of the systems is applied to itself. In the case of the legal system, the corresponding distinction is between legal and illegal; and what is determined as legal or illegal thereafter belongs to the system (Luhmann, 1993a, 67ff.). If, however, the distinguishing between legal and illegal—for instance by a court judgment—itself becomes the object of the question whether it is legal or illegal, then the legal system confronts its paradox: can law simultaneously be non-law, and non-law simultaneously be law (Luhmann, 1988)? The explosiveness of this paradox of law consists in the fact that, according to Luhmann (1993a, 546 f.), it cannot be definitively resolved (see also Corsi, 2016a, 12f). What matters, rather, is that the legal system deparadoxizes itself by operationally blocking the occurrence of its paradox and semantically invisibilizing it.

Deparadoxization succeeds when, with the aid of a constitution, a second distinction is introduced into the legal system: between constitutional law and other law. What is decisive is that constitutional law takes precedence over other law. Whether the distinguishing between legal and illegal is itself legal or illegal is thereby replaced by the “new” question of whether it is constitutional or unconstitutional. For this distinction not to appear as a question of law itself, which would merely shift the paradox elsewhere (Luhmann, 1993b, 294), the constitution must be seen as a political decision, rather than a legal one, made by the political constituent power.

For example, this occurred in 1995 in the well-known Crucifix Case in Germany (BVerfGE 93, 1). A family in Bavaria did not want their children to attend a school where crucifixes were displayed in the classrooms. However, the Bavarian school regulations required this practice. The family challenged the regulation in court. The paradoxical question of whether the legal norm, i.e., the school regulations, was itself illegal did not arise, however. This was blocked by the German constitution. Instead, the parents argued before the Federal Constitutional Court that the school regulations violated their fundamental rights and were therefore incompatible with the constitution (Brodocz, 2003a, 187ff).

From the perspective of the political system, which is coded according to power superiority and power inferiority (Luhmann, 2000, 88ff.; see Brodocz, 2012), the constitution, by contrast, simultaneously appears as supreme law. As a result, it is not a question of power superiority and power inferiority who is in fact power-superior or power-inferior, but instead a question of constitutionality (Luhmann, 2000, 391 f.). And this was also the case in the Crucifix Case, which was already mentioned (Brodocz, 2003a, 187ff.). Whether crucifixes were to be displayed in classrooms and whether the parents were legally obliged to comply with this requirement could not be determined by the Bavarian state government merely on the basis of its de facto superiority of power. Rather, what mattered was whether this power was compatible with the constitution. This is precisely what the Federal Constitutional Court denied. The parents’ constitutional complaint was successful.

By blocking the occurrence of the political paradox, the constitution thus performs on the side of the political system the same achievement as on the side of the legal system. Because both function systems simultaneously structure their expectations of one another by reference to the constitution, the constitution functions according to Luhmann (1990) as their “structural coupling.” Owing to this special form of structural coupling, special because it has a deparadoxizing effect, both systems moreover describe themselves as a unity, the “Rechtsstaat” or constitutional state governed by law (Luhmann, 1993a, 422), and in this way invisibilize their paradoxical constitution (Opitz, 2012, 104 f.).

Luhmann (1990) reconstructs this configuration of law, politics, and constitution historically on the basis of the constitutional history of the Western liberal constitutional state. Against this background, he also emphasizes that this configuration is not necessarily given with the differentiation of the political system and the legal system. In Luhmann’s systems theory, this contingency is systematically located on three different levels: systemic operations, systemic self-descriptions, and system differentiation.

At the *level of systemic operations*, contingency appears in the fact that the structural coupling of the legal system with the political system through the constitution is neither necessarily nor compellingly connected with both systems simultaneously deparadoxizing themselves with its aid and thereby stabilizing their operational differentiation. On the contrary, compared with other function systems such as the economy, science, or religion, the fact that these two systems reciprocally deparadoxize themselves through their common structural coupling is the exception (see Luhmann, 1997, 776ff.; Brodocz, 2003a, 192ff.; Brodocz, 2003b).

At the *level of systemic self-descriptions*, contingency is grounded in the fact that no semantics proceeds causally from the structure and mode of operation of the systems that describe themselves. For this reason, a self-description shared by the political system and the legal system is not necessary; systems coupled in this way can also describe themselves differently. The semantics of the Rechtsstaat in particular, as Opitz (2012, 105) notes, “has […] repeatedly proved to be extremely precarious.”

At the *level of system differentiation*, contingency is built into the fact that the functional differentiation of world society, which is required for the differentiation of operationally closed systems of law and politics, has not (yet) fully prevailed in all regions (Neves, 2006). Regionally, the societal system may, for example, still be differentiated primarily stratificatorily, that is, according to politico-economic strata (elite/people). How system differentiation then develops is closely connected with how law and politics stabilize themselves systemically and how they semantically refer to one another in the process. Thus, semantics may perhaps first develop that force the differentiation of the political system and the legal system (in the sense of Stäheli, 1998a), rather than merely representing it retrospectively as in Luhmann’s classic assumption (Luhmann, 1980).

Against this background, at least six different configurations of law, constitution, and politics can be hypothetically justified for Luhmann’s systems theory:

- “Symmetric coupling”: We use this term to describe the configuration of the European constitutional state as presented by Luhmann. Law and politics stabilize their differentiation as operationally closed systems by structurally coupling with one another operationally through the constitution and deparadoxizing themselves symmetrically through it at the semantic level.
- “Asymmetric coupling”: In the case of structural coupling, deparadoxization through the constitution can also occur unilaterally. In this configuration, either the political system alone deparadoxizes itself with the aid of its structural coupling with the legal system through the constitution, without this also occurring conversely on the side of the legal system; or the legal system deparadoxizes itself with the aid of its structural coupling with the political system through the constitution, while the political system does not use this option for deparadoxization. Structural coupling through the constitution thus does not provide the deparadoxization of both systems symmetrically, but asymmetrically.
- “Pure coupling”: Structural coupling between the legal system and the political system through the constitution is also possible without either of the two systems simultaneously using it at the semantic level for deparadoxization. Both systems solve their problems of paradoxization in another way. Without the deparadoxizing effect, structural coupling through the constitution in this configuration therefore appears as a pure variant.
- “Alternative coupling”: Both the political system and the legal system can differentiate as operationally closed function systems without being reciprocally structurally coupled through a constitution and simultaneously deparadoxized at the semantic level. This configuration is characterized by the fact that both systems possess functional equivalents to the constitution in order to couple structurally and to deparadoxize themselves; they are thus alternatively coupled.
- “Volatile coupling”: Given the process-theoretical nature of Luhmann’s systems theory, it is also reasonable to expect that the aforementioned configurations do not necessarily become permanently established in a manner that forms the structure of system relations but rather can be reconstructed as salient forms within a specific dynamic only for very short periods of time. In this variant, short phases of symmetric coupling can alternate with short phases of asymmetric coupling or alternative coupling. This variant is marked by the temporal instability of intersystemic relations, whereby law, politics, and the constitution find a configuration only for short phases because the couplings are highly volatile.
- “Dedifferentiation”: Due to the event-like character of the communications in which law and politics are processed, it is also possible that, at the level of system differentiation, law and politics in certain regions of world society do not differentiate stably into two different function systems at all. This would entail a configuration of law, politics, and the constitution in which no need for structural coupling or deparadoxization arises. The defining feature of this configuration would therefore be the dedifferentiation of law and politics.

### 3.2 Relevant cases: Bolivia and Ecuador

As Neves and Mascareño show, contemporary constitutional change in Latin America offers empirically especially interesting empirical cases for a systems-theoretical analysis of the variance in the configuration of law, politics, and the constitution. In research, this change has for some years been grouped under various labels such as “neo-constitutionalism” or “New Latin American Constitutionalism” (Alterio, 2021; Nolte and Schilling-Vacaflor, 2012; Tushnet, 2017). Despite the differences between individual cases (see King, 2013; Curcó, 2018: 213; Corrales, 2018: 13) Mc Manus (2022) identifies a common new feature in the constitutional developments in this region: a preference for an activated popular sovereign. This was regarded as the means for achieving greater social justice by enhancing popular sovereignty, whether articulated through the legislature, the executive, or a constituent assembly. Viciano and Martínez (2010, 2011, 2012) emphasize as a further feature that this constitutionalism signifies an overcoming of the nominalism that had characterized Latin American constitutionalism since its beginnings (similarly Mc Manus 2022; critically, for example, Couso 2013; Gargarella 2018; Madrid et al. 2010; Negretto 2009; Nolte 2017, 655). Within the framework of the rediscovery of the *pouvoir constituant* as the supporting pillar of the constitution-making process, citizens played a significant role in drafting and adopting constitutions (see also Corrales, 2018, 4). For Viciano and Martínez, this showed that a country’s constitution should not be reduced to a limiting function of power but should first be regarded as an expression of the basic political decisions of each people made in a constituent assembly. Another feature of ‘New Latin American Constitutionalism’ is that the constitutional text is shaped more by the experiences of grassroots movements than by experts and jurists (Viciano and Martínez, 2011, 18). Any inconsistency that might result from this would prove precisely the authenticity of the constitution-making process.

Although Venezuela is historically regarded as the point of departure for constitutional transformations in Latin America, Viciano and Martínez (2010, 25) point out early on that the Ecuadorian and Bolivian constitutions represent a variant differing from the Venezuelan case. The reason is that the changes in the field of fundamental rights, institutional innovation – for example in the form of the plurinational state – the synthesis of liberal and indigenous views, or the direct election of constitutional judges (in the Bolivian case) were far more radical than in Venezuela.

This is why we consider Bolivia and Ecuador to be the most relevant cases for examining three caesuras that are crucial for understanding the configuration of law, politics and the constitution in the context of this ‘New Latin American Constitutionalism’.

The *caesura of “plurinationality”* as an expression of the postcolonial constitutionalization of the political autonomy and participation of Indigenous peoples (Santos, 2012). In Bolivia, for example, four Indigenous territories were recognized as autonomous regions of the Bolivian state by 2020, following a lengthy review and participation process (Böhrt, 2020). This recognition is considered the territorial expression of ethnic groups that are now recognized as “nations”. In Ecuador, Article 1 of the new constitution explicitly states that Ecuador is a plurinational state, which Indigenous peoples, who were still regarded as deprived of rights in the 1970s (Lupien, 2011, 792), had already demanded in the early 1990s. This caesura is not about dealing with “national minorities” but with “minority nations” (Requejo, 2010, 54) within one state.

The *caesura of “the elevation of the people”* as the source of all state legitimation, both in the form of a latent *pouvoir constituant* and in the effort to hand over large parts of independent power control to the people. In Bolivia, the elevation of the people was formalized under the new constitution of 2009 through the hitherto unique democratic direct election of constitutional judges (Driscoll and Nelson, 2017; Pásara, 2015). The two electoral acts carried out so far, however, were marked by a high degree of nonparticipation (Driscoll and Nelson, 2019) and by the systematic subordination of weighty decisions by the directly elected constitutional judges to the power interests of the governing party. In Ecuador, by contrast, this elevation is evident in the fact that “the people” are considered the primary controllers of the state’s powers (Martínez, 2010: 27). Thus, the associated “function of transparency and social control” is regarded as the “most important innovation” of the Ecuadorian constitution of 2008 (Martínez, 2010, 27), and the organ provided for this purpose, the “National Council for Citizen Participation,” as the “main body” within the newly created control function (Grijalva, 2012, 33). In spite of this enhanced popular power, Wolff (2008) argues that in Bolivia the newly emerged type of democracy does not undermine the liberal tradition but complements it; something similar has also been put up for discussion with regard to Ecuador (Trujillo and Ávila, 2008).

The *caesura of “the legal subjectivation of nature”* (Berros, 2021; Gutmann and Valle, 2019), which was constitutionalized directly in the constitution of Ecuador and indirectly in the constitution of Bolivia. In Ecuador, the first judicial decisions have meanwhile been handed down within the framework of the rights of nature in application of Articles 71ff. of the 2008 constitution, beginning with the judgment in the well-known case of the Vilcabamba River in 2011 (Daly, 2012). In Ecuador, this constitutional development even led to the inclusion of “crimes against the environment, nature, or Pachamama” in the criminal code from 2014 onward, including “crimes against biodiversity and against natural resources, including water, soil, and air” (Kauffman and Martin, 2017). In Bolivia, the rights of nature are regarded above all as a socialist alternative to capitalism, as is evident, for example, in legal developments such as the Law on the Rights of Mother Earth of 2010 and the Framework Law on Mother Earth and Integral Development for Living Well of 2012 (International Rivers, Earth Law Center, Cyrus R. Vance Center for International Justice, 2020).

These three constitutional transformations – plurinationality, the elevation of the people, and the legal subjectivation of nature – require the relationship between law and politics in Bolivia and Ecuador to be reconfigured. This makes both cases especially informative for a systems-theoretical analysis of how law and politics differentiate, structurally couple and deparadoxize themselves under these conditions, and the function(s) that the constitution thereby assumes in law and politics.

### 3.3 Empirical research design: methods and data

As shown above, the contingency from which the variance of the six different configurations of law, politics, and constitution can be derived is systematically situated in Luhmann’s systems theory at the different levels of systemic operations, systemic self-descriptions, and system differentiation. Any empirical operationalization derived from this must therefore start with the research questions specific to each of the three levels:

-Level of systemic operations: In what form and with what “intensity” (Teubner, 2015, 84) do paradoxizing moments occur in political systems and legal systems in their constitution-making processes as well as in proceedings before their constitutional courts? Do both systems deparadoxize themselves reciprocally, as in the model of the Western liberal constitutional state? Are the constitutionalization of the elevated sovereignty of the people as a politics-immanent self-transcendence or the legal subjectivation of nature as a coupling with the international legal system used as alternative mechanisms by which the political and legal systems in Ecuador and Bolivia deparadoxize themselves? Or can other functional equivalents be found? Is the “global constitution” of human rights (Fischer-Lescano, 2005) used for this purpose and thereby re-provincialized? Which continuities and discontinuities can be reconstructed at this level?

-Level of systemic self-descriptions: Do the semantics of ‘New Latin American Constitutionalism’ offer Ecuador and Bolivia similar or entirely different self-descriptions of their political and legal systems to those offered by the semantics of the ‘Rechtsstaat’ that dominate liberal constitutional states and span both systems? Is “democracy” in “New Latin American Constitutionalism,” for example, a purely political self-description of the political system that does not refer at all to its structural coupling with the legal system, but represents a variant of self-transcendence in decolonial justice discourse? And is the semantics of the “Estado de Derechos” (state of rights), which is explicitly directed against the Western liberal idea of the *Rechtsstaat* (“Estado de Derecho”), an exclusively legal self-description of the legal system that does not refer to its structural coupling with the political system? Which continuities and discontinuities – for instance in the political semantics of the “sovereignty paradox” (Kastner, 2007) – can be reconstructed?

-Level of system differentiation: From the interplay of operation processes and semantic self-descriptions, conclusions can then be drawn about the form and degree of functional differentiation of the political system and the legal system. Does the semantics of ‘New Latin American Constitutionalism’, for example, make a retrospective or preparatory contribution to enabling politics and law to differentiate as autonomous function systems in the first place? Or can examples be found here of a political system and a legal system deparadoxizing themselves independently without structural coupling through a constitution? Do the political system and legal system deparadoxize themselves independently without mutual structural coupling through a constitution? Or are these cases examples of how political power concentrated in the elite is called into question by the institutionalization of another “power-superior” entity in the form of “the people”? Does it become apparent here how functional and stratificatory differentiation compete (Albert, 2016: 46ff.)? What function would the constitution have in such a case? The functional method, as outlined by Luhmann (2005a; 2005b), is a suitable overarching analytical strategy for answering these research questions. It examines the forms of operative “processing and unfolding of contingency” (Nassehi and Saake, 2002, 71) that can be traced in societal communication processes by means of the observational schema problem/solution. In doing so, it remains open to the description of functional equivalents by which similar problems can be processed in different ways (Luhmann, 1984, 84; Knudsen, 2010, paras. 8–9).

The analysis at the three levels requires different empirical data and methods. At the level of systemic operations, the focus lies on operative deparadoxization mechanisms by means of which paradoxizing moments in the legal system or political system are communicatively blocked, or gradually resolved, by reference to the constitution or functional equivalents. This requires empirical material in which paradoxizing moments are likely to occur – that is, moments in which the fundamental issues raised by constitutional changes concerning the distinction between legal/illegal in the legal system, or power superiority/inferiority in the political system, are discussed and negotiated. To trace how paradoxizing moments are operatively processed in political or legal communication (see Stäheli, 1998b, 61), one must focus on debates in the constitutional assemblies as well as on thematically relevant case law of constitutional courts. While constitutional debates explicitly renegotiate the fundamental principles of power attribution, constitutional law explicitly entails and, if necessary, readjusts the relationship between what is legal and illegal. In addition, parliamentary debates, committee meetings, or court hearings are relevant for the analysis of spontaneous communication. This is because these interaction systems allow to observe shifts among different function-systemic references, which is a common strategy for blocking paradoxes (see, for example, Jung, 2009; Bora, 1999).

Methodologically, Vogd’s contextural analysis is suitable at this level. It was developed to reconstruct precisely such “polycontextural arrangement[s]” of different system logics within interactive or organizational “composite contextures” (Vogd and Harth, 2019, para. 58; Vogd, 2011, 102). Contextural analysis is particularly useful because it can reconstruct how different system logics operate simultaneously. It allows researchers to identify how function systems deal with paradoxes. Such strategies include “structure-securing operations” such as changes of topic or markings of irrelevance (Bora, 1999: 97, 301 f.; Schneider, 1997), oscillations between interpretations (Kusche and Schneider, 2010: 10), and the emergence of new interpretive structures (Krönig, 2007). In the cases of constitutional reform in Bolivia and Ecuador, the concepts of plurinationality, the elevation of the people, and the legal subjectivation of nature have sparked lively debates in parliament and the constitutional courts. Since these debates focus on how new notions of nationhood, participatory democracy, and the rights of non-human entities are changing the way power and rights are allocated within the political and legal systems, they fundamentally challenge the ways in which politics and law have dealt with their paradoxes so far.

At the level of self-descriptions, the semantics of legal and political systems must be reconstructed. This entails analyzing how law and politics refer to one another semantically in the context of the ‘New Latin American Constitutionalism’, and the extent to which these references contribute to the invisibilization of systemic paradoxes. It also involves determining whether and what role the constitution plays in this, in its new, transformed form. To reconstruct these aspects of the political and legal systems’ self-descriptions as systemic theories of reflection (Kaldewey, 2013, 133), it is necessary to draw on central documents of constitutional discourse, such as legal and political-science commentary, as well as political commentary from major weekly newspapers, radio broadcasts, television programs, and social-media channels.

A discourse analysis following Keller (1997) is suitable for the analysis of these documents. Discourse analysis’ compatibility with systems theory has already been justified and tested both methodologically and empirically (Keller, 2010; Siri and Robnik, 2016; Weingart et al., 2002). Key passages in which central elements of the self-description of politics and law manifest themselves can additionally be analyzed in fine sequential detail by drawing on objective hermeneutics (on the methodological compatibility of objective hermeneutics and systems theory, see, for example, Mölders, 2011, 206 f.).

Based on the results of these operative and semantic analyses, then conclusions can be drawn for the level of system differentiation as to which variant of the configurations of law, politics, and the constitution is ultimately present in the cases of Bolivia and Ecuador. Examining how deparadoxization mechanisms emerge and become institutionalized allows to assess the long-term stabilization of legal and political systems. It also helps determine whether law and politics rely on each other, on the constitution, or on alternative mechanisms to manage their paradoxes: Do law and politics refer reciprocally to one another in order to block their respective paradoxes operatively, or do they use other systems for this purpose? Does the constitution play a role here? And what impact is to be assumed from the caesuras of plurinationality, the elevation of the people, and the legal subjectivation of nature? Drawing on the genesis and evolution of systemic self-descriptions, the current status of systemic differentiation can then be discussed: Do semantics such as those of ‘New Latin American Constitutionalism’ in Bolivia and Ecuador make a retrospective (Luhmann, 1980) or preparatory contribution (Stäheli, 1998a) to enabling politics through “democratization” and the legal system through “constitutionalization” to differentiate as autonomous function systems in the first place?

## 4 Discussion and conclusion

Against the background of the six hypotheses developed above concerning the variance of the configuration of law, politics, and constitution, it becomes clear that the approaches of Neves and Mascareño can be systematically distinguished as two different configurations of law, politics, and constitution. Neves assumes that functional differentiation has not prevailed in Latin America and claims that, for this reason, no system there is autonomous and operationally closed. Consequently, he also calls into question the role of the constitution as structural coupling of the legal system and the political system. Neves thus represents, for Latin America, the position that law, politics, and constitution have configured themselves there in the variant of “dedifferentiation.” Mascareño, by contrast, does not fundamentally call functional differentiation, and thus also the autonomy of the function systems, into question. Only in certain episodes is the autonomy of function systems suspended, without being permanently damaged. For this reason, unlike in Neves, the primacy of functional differentiation is preserved in Mascareño. The main trait of this development in Latin America is said to be the concentric structure of functional differentiation, from the center of which the political system repeatedly intervenes in the other function systems. Yet because, according to Mascareño and in contrast to Neves, the accompanying moments of dedifferentiation are more exceptions (“episodes”), for him the questions of structural coupling and deparadoxization remain unaffected by them. Mascareño’s approach can therefore be systematically summarized as an example in which law, politics, and constitution are configured in the variant of “volatile coupling.” It also shows very well for this variant that function systems need not necessarily become unstable as a result of this volatility. What seems decisive is that functional differentiation does not suffer permanently under the volatile coupling of law and politics. According to Mascareño, this becomes possible under conditions of a concentric development in which the political system remains at the center. Here the disturbances of other systems thus appear as an episodically recurring but also temporary pattern of instability (i.e., a kind of “punctuated equilibrium”).

As we have shown (see above, 3.1), beyond the variant of “symmetric coupling” represented by Luhmann and the two variants of “dedifferentiation” and “volatile coupling” presented in the approaches of Neves and Mascareño, three further variants for the configuration of law, politics, and constitution can be justified in systems-theoretical terms: the variant of “pure coupling,” when the constitution structurally couples law and politics as function systems, but deparadoxization does not occur through the constitution; the variant of “asymmetric coupling,” when the constitution structurally couples law and politics as function systems, but deparadoxization through the constitution is provided for only one of the two systems; and the variant of “alternative coupling,” when law and politics constitute themselves as operationally closed function systems, but are structurally coupled and deparadoxized not through the constitution, but through functional equivalents.

That Neves and Mascareño have introduced into the discussion variants whose focus lies on the question of the successful assertion of functionally differentiated systems may also be a consequence of their methodology and data. Both base their arguments predominantly on external scientific descriptions. Empirical analysis of systemic operations of the legal and the political system thus mostly remains outside their studies, so that a *reconstructive access* to this level (see above, 3.3) and to the practices and mechanisms situated there is lacking. The same largely holds for the level of systemic self-descriptions, since these are usually accessed through external scientific descriptions.

For future studies the theoretically grounded hypotheses and methodology presented above offer greater precision and analytical accuracy for these processes. They explicitly address three levels of systems-theoretical analysis—systemic operations, systemic self-descriptions, and system differentiation – and propose empirical methods and data that can be used to reconstruct them for any specific case. Addressing each of these levels separately enables a more comprehensive analysis of the dynamics of societal differentiation. In the case of Latin America, for instance, this approach could help to shed more light on the issue on which Neves and Mascareño disagree most. This is because the question whether systemic autonomy should be regarded as the dominant (Mascareño) or the exception (Neves) cannot ultimately be assessed at the levels of systemic differentiation and self-descriptions, which both authors focus on, alone but also requires a thorough analysis of concrete systemic operations.

With its three major constitutional transformations – plurinationality, the elevation of the people, and the legal subjectivation of nature – Bolivia and Ecuador as very relevant cases also offers empirically promising material (see above, 3.2) for moving analytically even closer to the theoretical variants and their empirical actualizations through which law, politics, and constitution are configured in Latin America. This approach can thus complement what Neves and Mascareño have already done with their pioneering studies.

The insights that can be achieved with this theoretical framework and its empirical operationalizability can also provide impulses for debates that extend beyond these analyses. For systems theory, it could become clear whether and, if so, how it can open itself to postcolonial perspectives (Xavier and Hewitt, 2021) and thereby overcome its bias in favor of the “Global North” (see Hayoz and Stichweh, 2018; Goncalves, 2017). Conversely, a new theoretical offering could be made to postcolonial studies, which are currently dominated by neo-Marxism and poststructuralism, if systems theory proves in this project to be sufficiently sensitive to difference in the variations with which law, politics, and constitution configure themselves in the Global South and North (see already Grizelj and Kirschstein, 2014; Goncalves, 2013). And last but not least, an interdisciplinary bridge could finally be built in this way to the current discussion of a “southern turn” in comparative constitutional law (Dann et al., 2020).

## Data Availability

The original contributions presented in the study are included in the article/supplementary material, further inquiries can be directed to the corresponding author.

## Footnotes

1 All German and Spanish quotations have been translated into English by us.
